# General practitioners` experiences promoting digital activity tracking for patients with type 2 diabetes: a focus group study

**DOI:** 10.1186/s12875-026-03182-z

**Published:** 2026-01-26

**Authors:** Marita Frøyen Fløtre, Siri Dalsmo Berge, Thomas Mildestvedt

**Affiliations:** 1 Department of Global Public Health and Primary Care, University of Bergen, Bergen, Norway; 2 General Practice Research Unit, NORCE Research, Bergen, Norway

**Keywords:** General practitioners, Digital activity tracker, Exercise prescription, Diabetes type 2

## Abstract

**Background:**

Physical activity is a cornerstone in treatment for patients with type 2 diabetes. In Norway, estimates show that less than 30% of all adults meet the recommendations for physical activity. Most patients expect their GP to support them in adopting a more active lifestyle. However, lifestyle changes are time-consuming, and GPs have expressed the need for specific tools to support their patients effectively.

**Aim:**

What are the experiences of GPs in implementing physical activity as a therapeutic intervention through digital activity trackers to patients with type 2 diabetes.

**Design and setting:**

Using semi-structured interview guide, we conducted a focus group study to explore GP’s experiences and perspectives on the use of digital activity trackers with Personal Activity Intelligence (PAI) to promote physical activity. Thirteen GPs and one GP practice nurse aged 38 to 64 years old and located at five different GP`s offices in the south of Norway participated in the study.

**Method:**

The GPs and the GP practice nurse were divided into four focus groups. The participants were recruited through the study DIGIPAS, social media and our network. All interviews were digitally recorded. Data were analysed thematically using systematic text condensation.

**Results:**

Three overarching themes, each comprising several related subthemes, emerged: (1) PAI as an objective measure of physical activity, (2) Activity trackers with PAI as a motivational tool, but not for everybody, and (3) Lack of supportive structures as a barrier for implementation of physical activity as treatment. A digital activity tracker served as a motivational tool for some patients, but the GPs reported technological challenges particularly among elderly patients. They also expressed a necessity for improved structural long-term support in promoting lifestyle changes, as well as more collaboration partners in general practice.

**Conclusions:**

A digital activity tracker may support healthy lifestyles for some patients, but successful implementation in general practice requires better usability, integration and governmental support.

## Introduction

Physical activity is a cornerstone in treatment for patients with diabetes type 2 [1]. For adults, including patients with diabetes type 2, it is recommended to do at least 150–300 min of moderate-intensity aerobic physical activity, or at least 75–150 min of vigorous-intensity aerobic physical activity per week [2]. In Norway, estimates show that less than 30% of adults and elderly in general meet the recommendations [3].

Most patients visiting general practitioners in Norway are physical inactive. More than 85% of these patients want to increase their physical activity level, and more than half of them would like help from their GP to achieve this goal [4].

GPs may avoid addressing physical activity due to limited knowledge of exercise as medicine, time constraints, and the absence of specific tools to offer patients [5]. Norwegian GPs also have varied experiences with collaboration on lifestyle changes with Healthy Life Centres, and these exist only in 65% of the municipalities [6].

Technology-based interventions and wearable activity trackers increase physical activity and have beneficial effects on important health-related outcomes in patients with chronic diseases [7, 8]. They are feasible and can increase motivation for physical activity in patients in a primary care setting [9]. Wearable activity trackers provide autonomy supportive feedback by encouraging self-directed choices through goal setting and feedback of goal accomplishment, self-monitoring and rewarding effort and improvement [10].

Personal Activity Intelligence (PAI) is a scientific metric to measure and monitor a person`s physical activity level, based on individual heart rate during exercise [11]. PAI is based on the intensity of physical activity rather than the number of steps taken or minutes spent, and it is not limited to specific types of activities. By continuously measuring heart rate and connecting the heart rate monitor to a free application, PAI can be easily tracked. PAI points are earned each time the heart rate increases, and individuals who consistently achieve 100 PAI or more per week over time have a significantly lower risk of premature death and cardiovascular diseases compared to others [11].

Activity monitors with PAI used in heart rehabilitation and exercise groups can motivate to structured physical activity for persons with overweight, diabetes type 2 and cardiovascular diseases [12–14]. However, studies exploring what experiences GPs have with an activity monitor is lacking.

The transtheoretical model of change describes behavioural change as a longitudinal and often step-wise process, where relapses are normal [15]. The GPs engage in different change processes, and their therapeutic approach can enhance long term changes [16]. In self-determination theory (SDT) the basic psychological needs of autonomy, competence and relatedness is necessary to facilitate long term motivation and engagement, and when satisfied yield enhanced self-motivation and well-being [17]. Autonomy supportive feedback and monitoring are demonstrated to be an effective behavioural technique [18, 19], a workstyle suitable for the GP setting.

The aim of this study is to explore the experiences of GPs in implementing physical activity as a therapeutic intervention through digital activity trackers to patients with type 2 diabetes.

## Methods

### Recruitment

The participants were recruited mainly through the study “Activity as medicine for diabetes, digital for patient and doctor” (DIGIPAS). DIGIPAS is a study conducted by a research group consisting of cooperation partners from Norwegian Sports College, The Diabetes association, NTNU, Exercise is medicine (EIM) and Memento Active. The aim of the DIGIPAS study was to implement PAI as an official measure of physical activity for patients with diabetes [20]. This project recruited doctors and patients and organized implementation and necessary education for the employees at the GP offices. The GPs were recruited pragmatically from practices located in the south‑western region of Norway. A designated contact person at each practice was approached directly to inquire whether the GPs at that site wished to participate. The GPs provided instruction and guidance to patients regarding the tool, assisting them with the setup of the activity tracker and associated application. The PAI score was self-monitored by patients and communicated to their GPs during follow-up visits to facilitate discussion and enhance motivation for physical activity. All patients aged 18 and older with type 2 diabetes were eligible for inclusion. Exclusion criteria included lack of motivation, critical aortic stenosis, unstable angina, decompensated heart failure, and terminal renal failure. The project loaned digital trackers to patients who did not already have one. Aside from a complimentary 6-hour course on Exercise as Medicine offered to the GPs, no additional incentives were provided to either patients or GPs to encourage participation. The intervention period lasted three months.

Sixteen GPs were advocating PAI through the DIGIPAS study, and ten of these GPs accepted to participate in our study. To increase the validity and relevance of the study, we aspired to recruit more participants. A post was made in a private Facebook group for GPs in Norway, inviting members who had experience with PAI to an interview. A universal gift card was offered as gratitude. Through our network, we also reached out to several GPs that were familiar with PAI and had applied it in their consultations, especially with patients with type 2 diabetes. One doctor was recruited through social media and three doctors through our network. These GPs did not borrow trackers from the DIGIPAS study, so they either had trackers available in their own offices or used the tool with patients who already owned a digital tracker or could borrow one from family members.

All participants were informed about the researchers` background, the aim and purpose of the study.

### Study design

The participants comprised of six women and eight men aged 38 to 64 years old. They worked at five different GP`s offices in the south of Norway, thirteen were GPs and one was a GP practitioner nurse. The doctors were all specialists in family medicine and had worked as GPs for an average of 14 years. The nurse was a diabetes specialist nurse and had 18 years of work experience.

The participants were divided into four focus groups, comprising of five, three, two and four people, respectively. All groups comprised GPs representing at least two separate GP`s offices. One interview was split in half with a focus group for the first half and then an individual interview with the nurse for the last part. The interviews were conducted from February 2024 until June 2024. Each group was interviewed digitally, with the interviews lasting approximately 60 min (58–68 min).

We used a semi-structured interview guide, and the interviews were conducted by a facilitator (MF) and an observer (TM/SDB). We encouraged discussions around subjects that were not a part of the questions in the interview guide and ensured that all participants could give their reflections around a theme. One interview guide was used for the first interview, and then slightly adjusted for the second and third interview to increase the relevance of the study. We also modified the interview guide again for the fourth interview to adjust it to the participants who were recruited from outside the DIGIPAS-study. The interview guide is shown in Table 1.

**Table 1:** Interview guide

**Interview guide**
Brief research project introduction
**What is the role of physical activity as a therapeutic intervention in your clinical practice as a GP?**
How do you address the topic of physical activity in consultations, and for which patient groups do you consider it relevant to raise? Which tools, resources or collaboration partners do you use? Do you have any clinical examples where the use of physical activity as a therapeutic intervention has either yielded positive outcomes or, on the contrary, was unsuccessful?
**What has been your experience in promoting PAI to your diabetes type 2 patients?**
Experiences with the collection and reporting of PAI? What barriers do you encounter as a general practitioner when promoting PAI to your patients? How does the use of PAI affect your motivation for using physical activity in the clinical treatment? How does PAI affect the patients’ motivation for being physical active? Can you give any clinical examples where the use of PAI have been successful, and examples where it has been unsuccessful?
**What are your thoughts on the future use of PAI in general practice?**
What are your thoughts on the implementation of an objective measure of physical activity, such as PAI, in current diabetes care? What factors could facilitate the use of PAI in clinical practice by GPs? Are there any other resources or tools that may help doctors to use physical activity as a therapeutic intervention? How do you think GPs can successfully help the patients to become more physical active?

Interview guide used in the focus group interviews with GPs about their experiences with promoting Personal Activity Intelligence (PAI) to patients with diabetes type 2.

### Data collection

All interviews were digitally recorded and transcribed directly after. The interviews were conducted through Microsoft Teams and recorded directly in addition to an external digital recorder as back up. Field notes were taken during the interviews. We used Microsoft Word for transcription of the sound files and corrected and anonymised the data in person afterwards. We ensured that all files were saved securely, and only software approved by the University of Bergen`s standards and recommendations was used.

### Data analysis

The interview data were analysed thematically using systematic text condensation described by Malterud [21]. This is a pragmatic method for cross-case analysis of qualitative data. All three authors read the transcripts independently before discussing and establishing preliminary themes. We then identified meaning units representing useful and interesting information around experiences with digital activity tracker and lifestyle changes in general practice. We coded and sorted the meaning units in subcategories under the themes. In this phase of the analysis, we used NVivo-12 to help identify and sort the meaning units. Through an iterative and inductive process, new themes and subcategories emerged. We contracted the meaning units to condensates, and through discussions we summarized the content to a generalized analytical text. We used key quotes to best illustrate our findings. The categories and sub-categories identified through the analysis process and representative quotes illustrating the findings are given in Table 2.

**Table 2:** Categories and sub-categories identified through the analysis process and representative quotes illustrating the findings

Categories	Subcategories	Quotes	Participants carachteristics
PAI as an objective measure of physical activity	Need for new tools	*The experience*,* really*,* is that it’s hard to change patients’ habits unless they want it themselves.*	Male 44
		*In my experience*,* supporting lifestyle changes is hard work*,* and only a few succeed. So*,* you end up thinking—maybe this is something other people should be doing instead.*	Female 43
		*Some patients appear really motivated during the consultation*,* but as soon as they leave the office the motivation seem to wander off.*	Male 39
	Specific and measurable	*It’s easier to recommend something quantifiable. It’s more specific and measurable. It gives the impression of us having something to offer.*	Male 48
		*People tend to find it fun when it’s something new*,* a bit competitive and concrete—and especially when you get a number to relate to. Many have immediately reacted positively and felt it was exciting to dive into something fresh.*	Female 44
		*It feels good to have something tangible to offer the patient when it comes to exercise. If I find the patient a little responsive it is nice to offer a tool to help them further on. And I find it motivational myself.*	Female 43
		*What motivates me to use PAI with patients is seeing that we can help them improve their health without relying on medication.*	Male 42
Activity trackers with PAI as a motivational tool, but not for everybody	Feedback on exercise	*PAI is very educational. Some patients say they train intensively*,* but don’t necessarily do that*,* and we get feedback that they can increase the intensity to achieve even better health outcome.*	Male 44
		*It’s typical when they’re at risk of starting blood pressure medication because their levels are a bit high. When I say*,* ‘If you exercise this much*,* it can lower your blood pressure by so-and-so*,*’ it’s kind of motivating—not having to take medication.*	Female 39
		*A number like that can be motivating*,* sure—but if we’re going to stick with it over time*,* I think people need to feel that it’s making a positive difference in their life overall. That they feel in better shape*,* feel better in themselves*,* sleep better*,* and so on.*	Male 68
	Not for everybody	*So clearly some patients just don’t get it*,* and the heart rate monitor is hanging loose like a big bracelet.*	Male 48
		*My previous job was in the district where the economic status was quite bad among many of the patients and where a small monthly expense made them sort of negative right away.*	Female 44
		*Interestingly*,* a few people I didn’t think would be into this really were. So*,* the experience was: don’t decide for others who might be up for it. Give them the chance to try.*	Female 50
Lack of supportive structures as a barrier for implementation of physical activity as treatment	Collaboration partners; Infrastructure and referrals	*Sometimes I have referred to a Healthy Life Center or recommended local activities*,* but it has not been systematic*,* rather more random.*	Female 50
		*I haven’t used green prescription or made a structured plan. I have just talked generally about physical activity.*	Female 43
		*The government pretends that they wish for more physical activity among patients*,* but they do not lift a finger to make it happen. There is no support*,* no incentives.*	Male 46
	PAI as a well-known motivational tool	*If we could use PAI more systematically it might become a part of the patient`s expectations as well. If we talk about it and use it*,* it will become a part of professional culture.*	Male 46
		*We do take 24-hour Blood Pressure*,* so maybe we can measure one week with physical activity by using PAI the same way.*	Male 64
		*I think it would be quite straightforward to introduce to everyone*,* if the time and technology were available. It allows us to explain that here you have a direct measure of health—of health benefits*,* physical activity*,* and exercise. If you manage to maintain 100 PAI*,* you will live longer and experience better health.*	Male 39

During the analysing process, we did not use any theoretical framework to guide the analysis. However, our results were discussed inspired by the Self-Determination Theory to further explore what experiences could foster motivation for the GPs in this field [17].

To improve the grammatical quality of the text in this study, Microsoft Copilot was used as a language editing and proofreading tool.

## Results

GPs considered digital activity trackers with PAI to be a helpful metric for implementing physical activity as a therapeutic intervention for some patients with type 2 diabetes in general practice. However, they encountered some technological challenges, particularly with elderly patients. The GPs reported a lack of structure and support in promoting lifestyle changes and few collaboration partners within general practice. The analysis resulted in three major themes: (1) PAI as an objective measure of physical activity, (2) Activity trackers with PAI as a motivational tool, but not for everybody, and (3) Lack of supportive structures as a barrier for implementation of physical activity as treatment.

### PAI as an objective measure of physical activity

Most GPs reported having discussed exercise with their patients. However, they described a lack of specific and structured tools for how to present and implement physical activity as part of clinical care. Working with lifestyle changes was described as demanding and complex, with successful outcomes observed in only a small number of their patients.

*“Some patients appear really motivated during the consultation*,* but as soon as they leave the office the motivation seems to wander off.” (male 39)*

However, one GP had noted that many patients use heart rate monitors and that GPs are often asked about metrics such as body battery and heart rate variability, which these devices measure. She had observed that there are many people in general that are interested in such concrete physiological indicators.

“*People tend to find it fun when it’s something new*,* a bit competitive and concrete—and especially when you get a number to relate to. Many have immediately reacted positively and felt it was exciting to dive into something fresh.” (female 44)*

The GPs found that PAI served as a simple metric for physical activity and it was easy to bring up and discuss during the consultations. They agreed that it was beneficial to have a tool that gave specific feedback. They could talk about exercise and health through an objective measure, and the doctors got a reference of what type and how much exercise the patients had done. Compared to a pedometer or patient reported activity, PAI felt more specific for the interviewed GPs.

“*It feels good to have something tangible to offer the patient when it comes to exercise. If I find the patient a little responsive it is nice to offer a tool to help them further on. And I find it motivational myself.” (female 43)*

One doctor felt particularly motivated upon realizing that she could help her patients achieve better health outcomes without relying on medication.

### Activity trackers with PAI as a motivational tool, but not for everybody

Another positive advantage with the metric was the patients` feeling of mastering and understanding. Several GPs mentioned that PAI gave the patient a certain understanding of the connection between physical activity and health. Some of the patients tended to believe their activity levels were reasonably good. However, when they began using the activity tracker and PAI, they discovered that their long walks gained few points due to low intensity.

Patients experienced that it was not dangerous to exercise with a high heart rate even with heart or pulmonary conditions. Those who were previously afraid to exercise now gained a better understanding of the exercise process, resulting in improved progression and a greater sense of achievement.

“*PAI is very educational. Some patients say they exercise intensively*,* but do not necessarily do that*,* and we get feedback that they can increase the intensity to achieve even better health outcome.” (male 44)*

One GP observed that activities she typically recommended to patients with joint pain, such as strength training and mobilisation did not contribute to any PAI. She emphasised the importance of informing patients that the benefits of these activities are substantial, even though they do not impact the PAI score.

The GPs experienced it was hard to introduce the digital activity tracker to those who were not used to exercise, and lack of achievement could affect the motivation. One doctor mentioned that there was a certain element of gamification and competition in PAI, which he found motivating for some patients, while others lost motivation when they failed to reach their goal. Although a numerical metric such as PAI may serve as a motivational tool for many individuals, the GP emphasized that maintaining long-term engagement requires experiencing tangible benefits of physical activity in everyday life.

“*A number like that can be motivating*,* sure—but if we’re going to stick with it over time*,* I think people need to feel that it’s making a positive difference in their life overall. That they feel in better shape*,* feel better in themselves*,* sleep better*,* and so on.” (male 68)*

PAI was also seen like a technical advanced tool. Some patients lost patience when they did not manage the technical part, such as connecting the monitor with the application. The GPs experienced that some patients needed a lot of information and guidance on the technical part and felt that introducing and explaining the activity tracker was time-consuming in a hectic workday. It was commonly perceived that there were challenges using the heart rate monitor and downloading and understanding the application on the smartphone, particularly among elderly patients.

“*So clearly some patients just don’t get it*,* and the heart rate monitor is hanging loose like a big bracelet.* “(male 48)

Another barrier mentioned was economy. Not all patients have a heart rate monitor themselves or can afford buying one.

However, one GP noted that it is difficult to predict what drives a patient’s motivation, and that one cannot know in advance who will respond positively to PAI without offering the opportunity to try it. While acknowledging the need to consider patients’ digital and technical capabilities, she emphasized that the tool should also be introduced to those who might initially seem less likely to engage. Several GPs shared positive experiences with patients they had not expected to benefit from PAI, but who began exercising regularly and achieved PAI scores beyond what the physicians had anticipated.

“*Interestingly*,* a few people I didn’t think would be into this really were. So*,* the experience was: don’t decide for others who might be up for it—give them the chance to try.” (female 50)*

### Lack of supportive structures as a barrier for implementation of physical activity as treatment

Many GPs shared the experience that collaboration in healthcare regarding lifestyle changes lacks a well-defined and effective structure. Some of the doctors referred patients to the Healthy Life Centre, while others provided advice on local community programs and associations. However, these recommendations were not given in a structured manner. Some GPs collaborated with physiotherapists when patients required rehabilitation or guided training for pain or injuries, but this collaboration did not extend to lifestyle changes.

Most GPs wished for a possibility to refer patients to a structured program with a good follow-up, and they agreed that the public arrangements for lifestyle changes should be organised in another way to reach out to more people. It was suggested to use the GP’s office as a centre and to have health- and exercise physiologists or physiotherapists to run lifestyle groups for the patients. Another GP stated that his motivation would have increased if he had experienced more help and assistance from the government, such as additional incentives for promoting lifestyle changes. He pointed out that Healthy Life Centres could work out well, but they exist only a few places and their capacity are limited.

“*The government pretends that they wish for more physical activity among patients*,* but they do not lift a finger to make it happen. There is no support*,* no incentives.* “ (male 46)

A digital activity tracker was not commonly used in general practice and the GPs experienced that few patients were familiar with PAI. If the GPs should use a digital activity tracker on a larger scale, they believed it would need to be more widely recognized by the public and something the patients expect to encounter during consultations. According to the GPs, their patients showed greater interest in well-known metrics such as blood pressure compared to activity measures. This affected the doctor`s motivation for promoting the latter.

Some GPs expressed a need for more support from the government. A digital activity tracker could be part of the applications offered by the Norwegian Directorate of Health. With an official recommended tool for treatment the GPs would not need to invest significant time explaining what it is and how to use it. Another GP proposed more external support, like referral of patients to a beginner’s course in digital activity tracking.

*“If we could use PAI more systematically*,* it might become part of the patients` expectations as well. By discussing and utilizing it regularly*,* it will become integrated into the professional culture.” (male 46)*

One of the GPs suggested an infrastructure that made activity tracking easier to apply throughout the health care and welfare system. He explained that if the Norwegian Labour and Welfare administration could use the metric as a demand for activity for every patient who is on work assessment allowance, then the metric would become more anchored in the system. Most GPs agreed that incorporating specific activity measures in the current diabetes care administrated by The Norwegian Organization for Quality Improvement of Laboratory Examinations (NOKLUS) could be beneficial, making it an expected measure for both patients and doctors. They suggested that the doctors could equip the patient with a heart rate monitor and control the application and software themselves. By doing this, the challenges with technology could be solved, and the GPs could evaluate how much health gain the patients would get from the activity done, one or two weeks at the time.

The GPs emphasized that, once PAI is established as a systematic health indicator and supported by appropriate structures and accessible technology, communicating PAI as a measure of health would be feasible.

*“I think it would be quite straightforward to introduce to everyone*,* if the time and technology were available. It allows us to explain that here you have a direct measure of health—of health benefits*,* physical activity*,* and exercise. If you manage to maintain 100 PAI*,* you will live longer and experience better health.” (male 39)*

## Discussion

### Summary

The GPs reported PAI to be a useful metric for promoting lifestyle changes in some patients with type 2 diabetes within general practice, though not universally effective. They found satisfaction in measuring physical activity using digital trackers and felt motivated to assist patients in achieving better health without relying on medication. However, they encountered challenges such as technological difficulties and the significant time required for implementation and follow-up. Additionally, the GPs highlighted a need for greater support, incentives and a more robust system for facilitating lifestyle changes in primary health care.

### Comparison with existing literature

This study shows that a digital activity tracker is a possible technological aid in lifestyle changes for patients with diabetes type 2 in general practice. This is in line with a pilot randomized controlled trial of 30 patients with type 2 diabetes in Quebec in Canada. They reported the implementation of an activity tracker in primary health care to be feasible and to improve cardiometabolic risk factors for patients with type 2 diabetes [22]. However, some problems remain to solve. In our study, GPs found it challenging to introduce an activity tracker to patients. In addition, technological difficulties like downloading software, login to the app and using the wearable activity tracker was an issue especially for the elderly patients. This is also pointed out as a potential barrier in a review from 2020 on published literature on digital health services providing cardiovascular care [23].

For a smoother introduction to patients, it was suggested to make the activity tracker a more widely recognized tool among both patients and GPs. Integrating PAI into the electronic medical record was mentioned, as it would also give a more structured surveillance of the patients` physical activity. An integrating of data from activity trackers has also been suggested in an intervention study from Lithuania, where 30 patients with impaired glucose levels used wearable activity trackers, as it is useful to get a clear picture of the patient`s overall health [24].

Motivation for adopting new treatment practices is essential for GPs. The lack of a structured system for lifestyle changes was a barrier for the GPs in introducing an activity tracker. In self-determination theory (SDT) the basic psychological needs of autonomy, competence and relatedness is crucial to facilitate long term motivation and engagement [17]. A structured system for lifestyle changes and support for using digital activity trackers could enhance GP`s sense of competence and, consequently, their motivation for practicing lifestyle medicine. With a digital activity tracker, the GPs felt they had a tangible tool to offer patients, making it easier to promote physical activity over medications. Self-monitoring of behaviour and goal setting are showed to be effectful techniques for lifestyle changes [19], which can support the use of a wearable activity tracker as a helpful utility. However, maintaining behavioural change is best supported through various other behavioural change techniques and a person-centred, autonomy-supported counselling approach [19], which underscores the need for a more structured system around lifestyle changes. The GPs found it hard to predict which patients would respond positive to the wearable digital activity tracker and make use of PAI. Prediction of change is a well-known challenge and even the best psychological theories for change will predict the variance of change in less than 1/3 of the cases [25].

The GPs report a lack of supportive structures in lifestyle medicine and describe a shortage of collaboration partners for promoting physical activity. This is in line with a cross-sectional study of 340 representative random GPs in the Netherlands. They reported little structural collaboration between GPs and exercise providers, because of barriers such as limited financial possibilities and restricted knowledge of local exercise facilities. However, when collaboration existed the GPs referred more often [26]. A Norwegian study of collaboration between GPs and the Healthy Life Centres from 2016 also showed that GPs have varied experiences, pointing out that it was only suitable for certain patients. It offered mostly day-time appointments, and there was no scientifically documented effect of the treatment. This was major obstacles for collaboration [6].

Some GPs reported that PAI was not a suitable measure in general practice because it emphasizes intensity and rewards those who exercise at a high heart rate. This is based on research indicating that small amounts of high-intensity training yield better fitness and health outcomes [11]. While World Health Organization`s guidelines “Every step count” emphasizes that all physical activity is good [2], mobility-training and low-intensity exercise like climbing, yoga and golf will often not lead to an increase of PAI. Therefore, when using PAI as the digital activity tracker, it is important to inform the patients about its focus on intensity and that PAI is primarily a useful tool for cardiac health.

A barrier to implementation identified by the GPs was the economic cost associated with digital activity trackers. Not all patients can afford such devices, which may consequently exclude some individuals from the intervention. It was proposed that GP offices could lend trackers to patients for a limited period, similar to the practice with 24-hour blood pressure monitoring. However, the application also enables individuals without heart rate monitors to measure PAI through manually registration of the activity.

Some GPs expressed a need for greater governmental support. A digital activity tracker could be included among the applications offered by the Norwegian Directorate of Health. With an officially recommended tool for treatment, GPs would not need to spend significant time explaining what it is and how it should be used. Given the wide range of activity trackers available, the government could also assist by identifying which mobile applications are CE-marked as medical devices.

### Strengths and limitations

Throughout the analysis process, we ensured that our conclusions were substantiated by cross-referencing the interview transcripts. This approach also enhanced the internal validity of our findings. The semi-structured interview guide was used as a guidance during the interviews, but the participants also mentioned interesting perspectives that needed further questions to clarify and elaborate. The conversation flowed smoothly among the participants, reducing the need for the interviewer to continually ask questions. Most of the participants knew each other from before. They all participated equally, and we had the impression that they all spoke freely and did not withhold any information. A limitation of the study is that participants were recruited both from a separate study and through our network, which may have introduced variability in their backgrounds.

The interviewers were GPs with a special interest in lifestyle medicine. To be a GP and have knowledge of the participants everyday practice is a strength, as it gives understanding of the possibilities and limitations in the participants` work. To strengthen the reflexivity during the analysing process we were aware that our background as GPs could make it more difficult to fully adopt an outside perspective on the participants’ experiences [27]. To mitigate this, we actively tried to adopt a reflexive approach, challenging our assumptions and remaining open to perspectives that differed from our own. Furthermore, we used a theoretical framework to better understand the results after the thematic analyse was finished.

A focus group design was chosen to facilitate discussions and interactions between the participants, aiming for more valuable data. This design allows for interaction, debate, and collective meaning-making, enabling participants to refine and expand on each other’s perspectives. Focus group interviews can therefore be a good method to collect experiences and perspectives among health care workers [28]. Additionally, homogeneous groups, such as GPs, appear to work more effectively than heterogeneous groups [29]. We aimed to recruit a varied study sample to ensure external validity and transferability of the results to other GPs. The participants were all experienced general practitioners. A limitation is that they were located in only two regions in Norway. However, they were both females and males in different ages, representing the general GP in Norway. The findings are not exclusively applicable to Norway but are transferable to other countries with a well-developed primary healthcare system and a high proportion of the population already using smartwatches.

The sample size was determined based on information power, which considers the amount of relevant information each participant provided, supported by high-quality dialogue, the focused aim of the study, and the specificity of the participants [30]. The sample size was fourteen persons, and after the fourth group interview, we had rich information about their experiences. There were few new discoveries that added further insights and we determined that we had reached an acceptable level of data saturation [31].

### Implications for practice and research

A digital activity tracker can be a valuable tool for some patients in general practice, and interested general practitioners can incorporate it into their practice.

Further research should investigate the effectiveness of digital activity trackers in general practice through controlled randomized clinical trials. Moreover, there is a need for clearer guidance on how to identify eligible patients for digital health interventions, as well as on determining which types of interventions are most suitable to integrate into general practice.

Additionally, new structures for collaboration on lifestyle changes in general practice are important to explore.

## Conclusions

A digital activity tracker can be a helpful tool for facilitating lifestyle changes for some patients in general practice, although it is not suitable for everyone. For such tools to become an integrated part of Norwegian general practice, technological and usability challenges must be addressed. GPs also call for greater governmental support and improved systems for collaboration to promote lifestyle change in primary healthcare. While these findings are grounded in the Norwegian healthcare context, they are likely transferable to other countries with a well-developed primary healthcare system and a high proportion of the population already using smartwatches.

## Data Availability

The data collected and used in this study cannot be shared publicly due to ethical reasons and the privacy of the individuals who participated in the study but are available from the corresponding author on reasonable request.
